# Paracellular Pathway Enhancement of Metformin Hydrochloride *via* Molecular Dispersion in Span 60 Microparticles

**DOI:** 10.3389/fphar.2019.00713

**Published:** 2019-07-18

**Authors:** Omar Y. Mady, Ahmed A. Donia, Adam A. Al-Shoubki, Waseem Qasim

**Affiliations:** ^1^Department of Pharmaceutical Technology, Faculty of Pharmacy, Tanta University, Tanta, Egypt; ^2^Department of Pharmaceutical Technology, Faculty of Pharmacy, Menoufia University, Menoufia, Egypt; ^3^Department of Pharmaceutics, Faculty of Pharmacy, Omar Al Mukhtar University, Albayda, Libya; ^4^Al Zahraa Hospital, Wasit, Iraq

**Keywords:** Span 60 metformin HCl, melt congealing technique, total permeability percentage, paracellular pathway, non-everted sac

## Abstract

Surfactants are well known as permeation enhancers. Span 60 microparticles encapsulating different concentrations of metformin HCl were prepared by using rapid congeal melting technique. Electro-scanning microscope showed smooth surface but less round microparticles. The actual drug content was nearly equal in the different particle sizes of the microparticles. Differential scanning calorimetry results indicated the molecular distribution of the drug molecules with no evidence of drug thermal degradation. The drug release profile from the microparticles has, in each case, burst and there was incomplete drug release. The drug partition coefficient is markedly enhanced as a result of its molecular dispersion in Span 60, indicating the increasing of the drug lipophilicity as a result of its encapsulation in the polar part of the surfactant. Non-everted sac was used to study the drug permeability after solving its critical points. Compared to pure drug, the permeability profile of the drug increased from the Span 60-encapsulated drug, with a total permeation of 68% and drug absorption enhancement of 253%. The drug permeation enhancement mechanism was suggested to be molecular dispersion in the matrix, which is emulsified by Tween 80, and this leads to increasing the hydrophilic paracellular pathway of the drug. Considering the emulsification system of the GIT, which emulsifies the Span 60 instead of Tween 80, a huge improvement of the biopharmaceutics classification system class III permeability and consequently bioavailability could be expected. In addition, this study will open the door to the use of the same technique for enhancing the drug absorption mechanisms by the paracellular pathway for rapid and complete pharmacological effect.

## Introduction

A congealable disperse-phase encapsulation technique was used for the preparation of animal fats or fats of vegetable origin as matrices (Hassan et al., 1995). The technique is seemingly for hydrophobic drugs and has good absorption rates (Adeyeye and Price, 1991; Adeyeye and Price, 1994; Edward et al., 2002; Gowda et al., 2014). Amidon et al. classified the pharmaceutical active ingredients into four classes based on the solubility and the intestinal permeability parameters. That is because the main factors, which manage the rate and extent of drug absorption, are its solubility and intestinal permeability (Amidon et al., 1995). Class one is all drugs which have high solubility and high permeability. The drugs of class two have high permeability but have also low solubility. Class three are the drugs which have high solubility and low permeability like metformin HCl. Class four drugs are characterized by their low solubility and low permeability. Self-micro emulsifying drug delivery systems (SMEDDS) are one of the techniques used to advance the drug solubility of class two. It is a pre-concentrated mixture of surfactants, co-surfactants, and lipophilic phase. This mixture forms fine droplets of emulsion with size range 5–100 nm when mixed with water or with the body fluids in the aqueous lumen of the gastrointestinal tract (Pouton, 1985a; Pouton, 1985b; Constantinides, 1995; Humberstone and Charman, 1997; Pouton, 1997; Pouton, 2000; Ghosh and Murthy, 2006). Metformin hydrochloride is, according to BCS, a class III drug, having high water solubility and low permeability through intestinal mucosa. Accordingly, any increasing of the permeability of the drug will lead to increasing its bioavailability (Singh et al., 2009) and consequently decreasing the daily dose. It was reported that the intestinal permeability of metformin HCl was enhanced by using a self-emulsified drug delivery system [SEDDS], which leads to increasing its oral bioavailability (Pouton et al., 1987; Wakerly et al., 1987; Craig et al., 1993; Garrigue et al., 2006; Patel et al., 2008).

Sorbitan monostearate (Span 60) is a synthetic wax of an ester of sorbitan (a sorbitol derivative) and stearic acid. It is a primary emulsifier and is allowed to be used as food emulsifier by the European Union (Current EU approved additives and their E Numbers, Food Standards Agency, 2011). In addition, there is no reporting about the carcinogenic activity of Span 60 at different dose heights in humans (Hendy et al., 1978). Mady (2017) succeeded in preparing Span 60 microspheres containing Ibuprofen by using solvent evaporation and melting congealing techniques. A comparison between the two techniques used for drug encapsulation was done. In vitro characterization of the products concerning the drug entrapment methods and drug release was also carried out. The results showed a promising encapsulation technique in the pharmaceutical technology. Accordingly, the aim of this work is to try to encapsulate metformin HCl as an example for class III in Span 60 as a drug encapsulation matrix and to study the role of the surfactant matrix in the enhancement of the permeability of a poor permeable drug of class III.

## Experimental

### Materials

Metformin Hydrochloride (HCl), El-Nasr Pharmaceutical Chemical Co, Egypt, Polyvinylpyrrolidone (PVP) 40,000 of research grade was gifted from Sigma-Aldrich Chemical Co. (USA). Span 60 of research grade was gifted from Atlas Chemise, IC GmbH (Germany). All other chemicals were of analytical grade and were used as received.

### Equipment

DSC (PerkinElmer simultaneous thermal analyzer SAT 6000, supplied with the following software: Pyris SAT 6000, USA), an electron microscope (JEOL-model: JSM-5200LV, Japan), a magnetic stirrer (VELP scientifica, Europe), a UV/visible spectrophotometer (Thermo Fisher scientific, model EVO 300PC, software: vision pro, USA), a vibrating set of sieves (VEB/letalweberei Neustadt, Orla, DD), and a Paddle USP dissolution apparatus, Type Dis 6000 (Copley scientific, UK) were used.

## Methods

### Preparation of Span 60-Metformin HCl Microparticles

Span 60 microparticles containing (25%, 33.33%, 50%, 66.66%, and 75%) theoretical drug content (TDC) were prepared using the melting congealing method (Mady, 2017). The required amounts of Span 60 and metformin HCl were weighed and melted. Then, the homogenous melting of the Span 60 was poured into 100 ml 0.1 HCl containing 3 gm PVP 40000 while stirring. The aqueous phase was either preheated to 60°C or used at room temperature. Stirring continued for either 20 min or until the system reached room temperature. The products were collected by filtration and air-drying.

### Characterization of the Microparticles

#### Electron Scanning Microscope Examination

The morphology, surface and internal structure of the prepared Span 60 matrices encapsulated metformin HCl were studied by using an electron-scanning microscope (SEM). The magnification used was done according to the morphology of the product and the best image that clarifies the surface shape and method of drug entrapment.

#### Sieve Analysis

Sieve method was used for the determination of the microparticles’ mean particle size (Gupta et al., 2009). A definite weight of microparticles products containing different percent of drug was placed on a set of standard sieves. The set of sieves was automatically vibrated for 10 min using a mechanical sieve vibrator at constant speed. The part of the microparticles remained on the sieves was weighed for the determination of the particle size distribution (El-Helw et al., 2008). The mean microparticles diameter was calculated using the following equation (Behera et al., 2008)

$$\;\begin{array}{l} \mathrm{Mean}\;\mathrm{particle}\;\mathrm{size}\\ \;=\frac{\Sigma \left[\mathrm{mean}\;\mathrm{particle}\;\mathrm{size}\;\mathrm{of}\;\mathrm{the}\;\mathrm{fraction}\;\times \mathrm{weight}\;\mathrm{fraction}\right]}{\Sigma \left[\%\mathrm{weight}\;\mathrm{fraction}\right]} \end{array}$$

#### Thermal Analysis

Thermal analysis of metformin HCl and different particle size of metformin HCl encapsulated in Span 60 was carried out. The heating cycle ranged from 20 to 240°C to cover the melting point of the pure drug, which is reported to be at 225°C (Bretnall and Clarke, 1998). Differential scanning calorimeter (DSC), differential thermal analysis (DTA) and thermal gravity (TG) were conducted for each sample simultaneously.

#### Determination of the Actual Drug Content

Metformin HCl method of analysis was standardized in 0.1 N HCl at 232 λ max using UV spectrophotometer (Mao et al., 2008). Then, 100 mg of the selected sieve fractions of each product was dissolved in 100 ml of 0.1 N HCL at 60°C. The resulting solution was measured at 232 λ max using 0.1 HCl as a blank. Sometimes dilutions may be done and the procedure was carried out in triplicate. The mean actual drug content (ADC) and encapsulation percentage were calculated using the following equations (Mao et al., 2008).

$$\begin{array}{l} \mathrm{Theoretical}\;\mathrm{drug}\;\mathrm{content}\;(\mathrm{TDC})\;=\frac{\mathrm{drug}\;\mathrm{total}}{\mathrm{drug}\;\mathrm{total}+\mathrm{span}\;60}\times 100\\ \mathrm{actual}\;\mathrm{drug}\;\mathrm{content}\;(\mathrm{ADC})\;=\frac{\mathrm{actual}\;\mathrm{drug}\;\mathrm{content}\;\mathrm{total}}{\left(\mathrm{drug}\;\mathrm{total}\;+\;\mathrm{span}\;60\right)}\times 100\\ \mathrm{Drug}\;\mathrm{encapsulation}\;\%=\frac{\mathrm{Actual}\;\mathrm{drug}\;\mathrm{content}\;\mathrm{total}}{\mathrm{Theoretical}\;\mathrm{drug}\;\mathrm{content}\;\mathrm{total}\;}\times \text{100} \end{array}$$

#### Experimental Determination of n-octanol-water Partition Coefficients [log P]:

A total of 20 ml of n-octanol containing 20 mg either of pure drug or of each product of the drug encapsulated in Span 60 and containing 20 mg metformin HCl determined from the actual drug content was taken. Then, 20 ml of distilled water was added while stirring. The formed system was transferred into a separating funnel and allowed to equilibrate. The concentration of the drug diffused from the organic phase (n-octanol) to the aqueous was measured spectrophotometrically at 232 λ max.

$$\mathrm{Log}\text{ p}=\log\left[\frac{\mathrm{solute}\;\mathrm{unionized}\;\mathrm{in}\;\mathrm{octanol}}{\mathrm{solute}\;\mathrm{unionized}\;\mathrm{in}\;\mathrm{water}}\right]$$

#### Studying the Drug Release Profile:

An accurate weight of the prepared Span 60 microparticles containing 500 mg of Metformin HCl (calculated according to the determined actual drug content) using particle size fraction (500–315 µm) was added to the USP paddle dissolution apparatus (Pharmacopoeia, 2007). The release solution was 900 ml of 0.1 N HCl with a maintained temperature at 37 ± 0.5°C and a stirring rate of 100 rpm. Samples of 5 ml were taken at predetermined time intervals, and the new release medium was added to replenish each sample withdrawn. Sometimes dilutions may be carried out, and the resulting solutions were measured at 232 λ max using 0.1 N HCl as a blank. The procedure was carried out in triplicate.

### Non-Everted Sac Model as a Tool to Evaluate Intestinal Permeability

#### Preparation of Non-Everted Intestinal Sacs

The experimental protocol was approved by the Faculty of Pharmacy ethical committee. The study used a male albino rabbit with a weight of 2 kg obtained from Tanta Animal House. The rabbit was accommodated in a clean room with free access to food and water. The rabbit fasted overnight with free access to water and was anesthetized by intramuscular injection of ketamine HCl. Upon confirmation of loss of the pain reflex, the animal was scarified. A midline longitudinal incision of 3–4 cm was made and the small intestine was located. A segment of the upper small intestine (14 cm) was used to prepare the sac. The lumen of the intestinal segment was washed with buffer to remove any solid material. Using a surgical thread, a side of the segment of the small intestine was tied. The fresh intestinal sac was then filled with buffer, the other side being tied with surgical thread and checked for leaks (Dudeja et al., 1995; Sakai et al., 1997).

#### Preparation of Perfusion Solutions

Different perfusion solutions were prepared and used for studying the drug permeation mechanism from the dosage form:

1. **Perfusion solution A:** An amount of Span 60 microparticles containing 50 mg of Metformin HCl as an actual drug content was accurately weighed. The weighed microparticles were selected to be with a particle size fraction range of 500–315 µm. The sample was dispersed in 4 ml of pH 6.8 phosphate buffer and then 1 ml of Tween 80 was added.
2. **Perfusion solution B:** The procedure is similar to step A but the intestinal segments were everted and placed in 10 ml chloroform containing 300 mg phosphatidylcholine. The segments were turned until complete evaporation of the solvent, and the everted segments were then turned again to be non-everted segments. They were then filled with the perfusion solution as in step A.
3. **Perfusion solution C:** This procedure resembles step A, but before dissolving the sample, an amount of pure Span 60 was calculated and added as a physical mixture to complete the amount of the surfactant to be 75% surfactant for the entire formula. Then, the physical mixture of the microparticles containing 50 mg drug and the added pure Span 60 were dispersed in 4 ml of phosphate buffer pH 6.8. After that, 1 ml of Tween 80 was added.
4. **Perfusion solution D:** This procedure is similar to step C, but before dissolving the sample, after the addition of the calculated amount of pure Span 60 to prepare 75% surfactant, the physical mixture was melted, cooled and then the produced mass was dispersed in 4 ml of pH 6.8 phosphate buffer. A volume of 1 ml of Tween 80 was added.

#### Drug Transfer in Non-Everted Intestinal Sacs

The fresh intestinal sac segment was emptied from the buffer solution. The intestinal sac was then filled with prepared perfusion solution (A, B, C or D), tied with surgical thread and tested for leaks. The segment length and diameter were measured for surface area determination.

#### Drug Permeation Profile Study

The prepared segment was suspended on the shaft of the USP dissolution apparatus. The outside of the sac medium (permeation medium) was 900 ml of phosphate buffer (pH 6.8) with a temperature maintained at 37 ± 0.5°C and stirring rate of 50 rpm with continuous aeration. Samples of 5 ml were taken at predetermined time intervals and the new release medium was added to replenish each sample taken. The amount of drug permeated from the segment to the medium was determined spectrophotometrically at 232 λ max.

#### Determination of Permeability Coefficient:

The permeability coefficient (apparent permeability) was determined by using Fick’s law across the isolated rat intestine (Sten-Knudsen, 1978; Page and Carlson, 1991). The law is described mathematically as

dM/dt=PS[Cd−Cr]

where dM/dt is moles of solute transported per unit time, P is the permeability coefficient, S is surface area of the membrane, Cd is the concentration of solute in the donor (serosal) phase and Cr is the concentration of solute in the recipient (mucosal) phase. In this case, the sink conditions prevail since the volume of the serosal fluid is much larger than the mucosal volume, then Cd is constant and much larger than Cr and Cr could be ignored. Accordingly, a simplified equation could be written follows:

dM/dt=PSCd

The variables M and Cd could be determined by analysis of mucosal fluid. The surface area (S) could be calculated by considering the intestinal sac a cylinder. Then, M/SCd could be calculated and plotted against time. The slope of the linear part of the plot is the permeability coefficient (P), which has the units of velocity (cm/s). The slope of the linear part of the curve was determined by linear regression (Sten-Knudsen, 1978; Page and Carlson, 1991). Another method for calculation of apparent permeability was also reported (Mateer et al., 2016). The author reported the procedure as follows, and the time should be converted to second units. For each time point, cumulative concentration (Q) is calculated from the following equation:

Qt=(Ct ×Vr)+(Qt sum ×Vs)

Where Qt is the cumulative concentration at time t, Ct is concentration at time t, Vr is volume at receiver side, Qt sum is the sum of all previous Qt and Vs is volume sampled. Then Q is plotted versus time (t) and the slope is calculated: δQ/δt. The apparent permeability (Papp) could be calculated using the following equation: Papp = (δQ/δt)/(A × Co), where A is area of tissue and Co is the initial concentration.

## Results and Discussion

Span 60 was prepared as a microsphere matrix containing ibuprofen by two methods, the solvent evaporation technique and the melting dispersion technique (Mady, 2017). On trying to encapsulate metformin HCl by Span 60, solvent evaporation could not be applied due to the insolubility of the selected drug in the organic phase. Accordingly, only melting dispersion was used. To prepare rounded microspheres by using a normal method, it is essential to preheat the aqueous phase to over the melting point of the matrix used before adding the melted Span 60 containing the drug and continuous stirring at room temperature until the product formed. This may promote the slow formation of the microsphere matrix.

**Figure 1:**
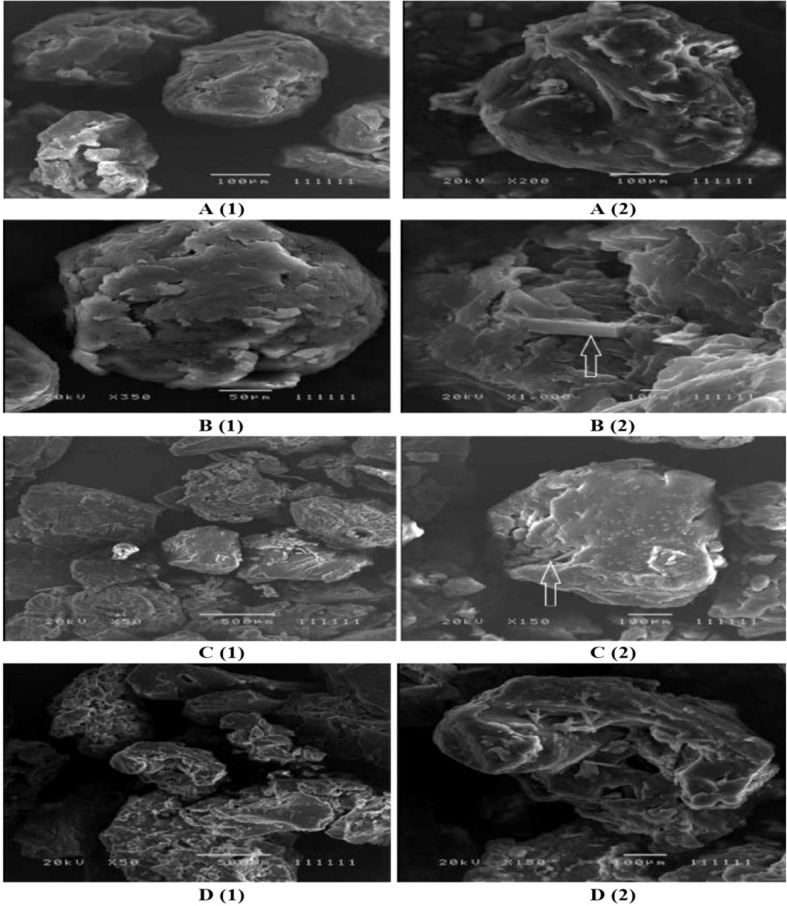
Electron scanning microscope photograph (ESM) of Span 60 microparticles containing drug: **(A)** (1&2) 25% TDC & normal method. **(C)** (1&2) 25% TDC & rapid congealing matrix. **(B)** (1&2) 75% TDC & normal method. **(D)** (1&2) 75% TDC & rapid congealing matrix.

**Figure (1, A1, B1)** shows the nearly round microspheres prepared by using 25% and 75% TDC or 75% and 25% Span 60, respectively. Increasing the magnification shows smooth surface microparticles (**Figure 1**, A2, B2). In addition, the presence of drug crystals entrapped in the Span 60 matrix (**Figure B2**) can clearly be noticed. **Table 1** shows a very low actual drug content (ADC) in the products prepared by the normal method. These results could be explained according to the diffusion of the drug from the melted emulsified Span 60 droplets containing drug to the aqueous phase as a result of high drug solubility in the aqueous phase used. This may also explain the nearly equal ADC in the microspheres prepared by using different TDC. To address the low drug entrapment issue, a rapid congealing of the melted phase would be applied by pouring the melted phase to non-pre-heated aqueous phase. The rapid congeal of the melted Span 60 containing drug, as a result of using the aqueous phase at room temperature, led to morphological change of the product and also ADC. **Figure 1** (C1, D1) shows the closing of the nearly round microsphere product and the formation of microparticles. Increasing the magnification shows the smoothness of the microparticles’ surfaces and the drug crystals entrapped in the Span 60 matrix can also be clearly noticed. **Table 2** shows the role of the rapid congealing of the Span 60 as a matrix on the hindering of the diffusion of the dissolved drug to the aqueous phase which would be reflected also on the ACD. In addition, the shock temperature changes of the drug from the melted state in the Span 60 matrix to room temperature when added to the aqueous phase should be also considered since the drug has a high melting point. This explanation could be supported by the closest of ADC in the different particle sizes of the product prepared by the same TDC and also from the values of standard deviations. From the same table, it can also be noticed that ADC is increased by increasing TDC, but in every case is lower than that reported by the author on using ibuprofen (Mady, 2017). That is due to the solubility and insolubility of metformin HCl and ibuprofen in the aqueous phase used respectively. The decrease in the drug encapsulation % by increasing TDC may be due to low ADC entrapped in the Span 60 matrix product compared to TDC used. That may be high drug solubility in the external phase. It should be also reported that the use of external phase, in which the drug has lower solubility, which is a recommended method to increase the drug entrapment percent, is not available in this case.

**Table 1:** ADC in the microspheres prepared by normal method.

TDC	AD C	± SD
25%	3.091	0.156
33%	3.321	0.203
50%	3.818	0.198
66%	4.100	0.179
75%	5.220	0.213

**Table 2:** ADC in the products prepared at room temperature [rapid congealing of the matrix].

TDC	item	550 µm	407.5 µm	210.5 µm	85.5 µm	Mean	Encap.%
25%	ADC ± SD	18.225 0.249	17.891 0.315	18.255 0.199	18.545 0.308	18.229 0.268	72.916
33%	ADC ± SD	18.255 0.795	20.218 0.844	19.345 0.797	19.273 0.812	19.273 0.803	57.876
50%	ADC ± SD	25.309 0.75	26.691 0.798	26.909 0.749	26.982 0.786	26.473 0.823	52.945
66%	ADC ± SD	27.073 0.741	27.564 0.807	27.891 0.763	28.891 0.766	27.855 0.768	40.319
75%	ADC ± SD	29.455 0.507	29.527 0.522	29.891 0.460	30.545 0.469	29.855 0.499	39.806

**Figure 2:**
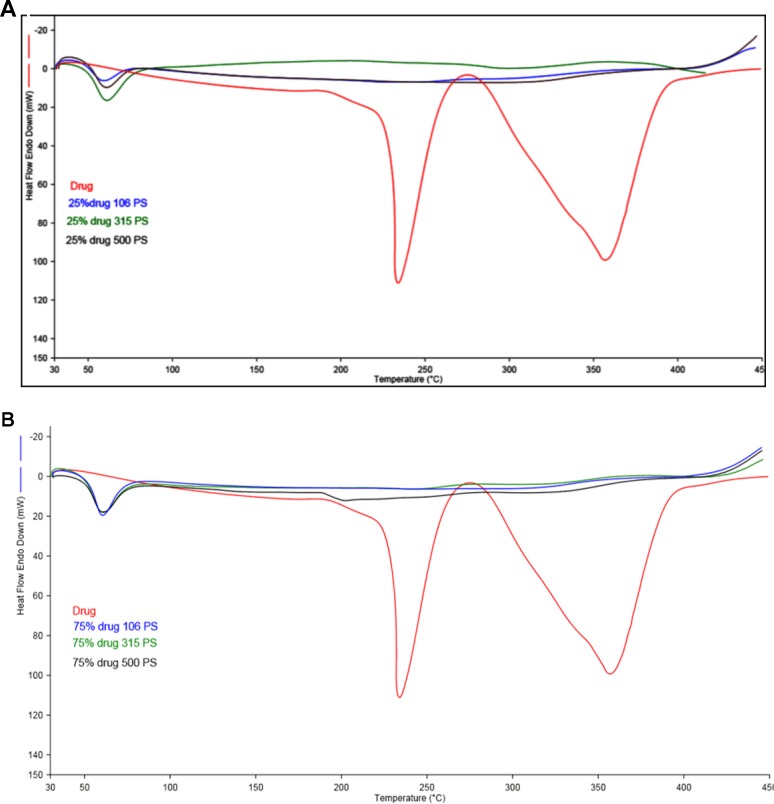
**(A)** DSC scan of different particle size of Span 60 microparticles containing drug prepared by using 25% theoretical drug content (TDC). **(B)** DSC scan of different particle size of Span 60 microparticles containing drug prepared by using 75% TDC.

**Figure 3:**
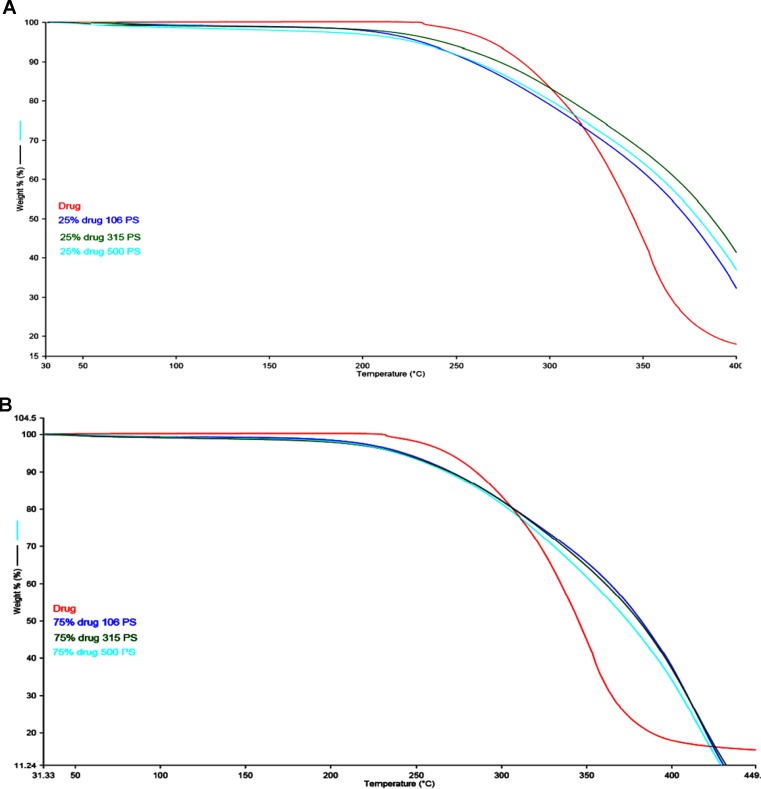
**(A)** TGA scan of different particle sizes of Span 60 microparticles containing drug prepared by using 25% TDC. **(B)** TGA scan of different particle size of Span 60 microparticles containing drug prepared by using 75% TDC.

**Figure 2** (**A**, **B**) represents the DSC thermogram of the pure metformin HCl and different particle sizes of Span 60-encapsulated drug prepared by using different TDC. From the figure, it can be noticed that DSC thermogram of pure metformin HCl exhibits a clear endothermic transition stage with a maximum at 234.6°C. This endothermic transition stage is attributed to the melting of the pure metformin HCl (Bretnall and Clarke, 1998). A huge endothermic peak is also observed directly following the melting of the pure metformin HCl, which is due to the decomposition of the pure drug (Plata-Vargas et al., 2017). More details about the thermal degradation of metformin HCl were reported by Ramachandran et al. (Ramukutty et al., 2014). These results are supported by TG analysis of the pure drug. TG thermogram of metformin HCl (**Figures 3A, B**) shows no weight loss attributable to loss of solvent, but does reveal noteworthy weight loss at about 256°C, which is due to the thermal breakdown of the compound (Bretnall and Clarke, 1998). The DSC of all different particle sizes of Span 60-encapsulated drug prepared by using different TDCs showed an endothermic peak at around 60°C. This endothermic peak represents the melting point of Span 60. At the same time, the DSC thermogram of different particle sizes of Span 60-encapsulated drug prepared by using 25% or 75% TDC showed a complete disappearance of all drug crystal endothermic patterns, indicating the molecular state entrapment mechanism (**Figures 2A, B**). The presence of a small broad event falls in the same position of the pure drug in the DSC scan of Span 60-encapsulated drug with a particle size of 500 µm prepared by using 75% TDC, indicating the presence of some drug crystals (**Figure 2B**). These results are in agreement with the results of the electron scanning microscope of the same product. The absence of drug melting endothermic peak as a result of its molecular dispersion in the Span 60 matrix did not indicate the role of the matrix in the drug thermal stability (Plata-Vargas et al., 2017). At the same time, a TGA thermogram of all Span 60-encapsulated drugs showed a delayed degradation effect compared to the pure drug (**Figures 3A, B**). That may indicate the enhancement effect of Span 60 against the thermal degradation of the drug.

**Figure 4:**
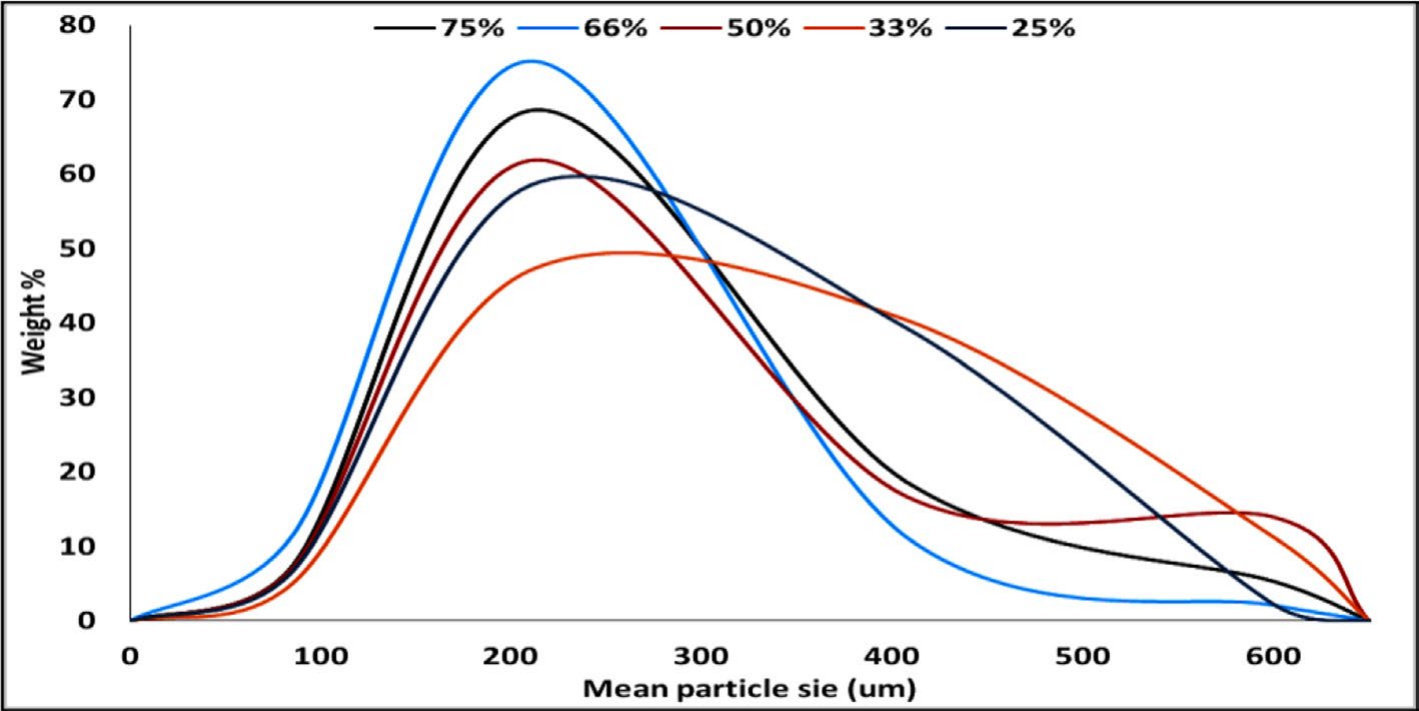
Particle size distribution of Span 60-encapsulated drug prepared by using different. Different TDC.

The particle size distribution curve showed a unimodal except on using 50% TDC which looks like bimodal one. The bell shape is clearly improved by increasing the TDC (**Figure 4**). The disturbance in the calculated mean particle size from low to high then low and latterly high on using 75% TDC (**Table 3**) may be due to the rapid congealing method used, the molecular state drug entrapment mechanism, big difference in the melting point of the drug and Span 60 and also the presence of some drug crystal on using 75% TDC.

**Table 3:** Mean particle size of the prepared products using different TDC.

TDC	Mean particle size
25%	286.784
33%	305.767
50%	281.763
66%	224.995
75%	255.719

**Figure 5:**
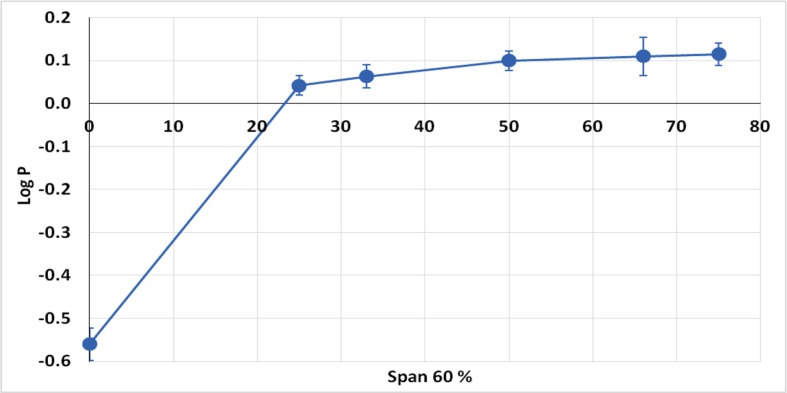
Partition coefficient of metformin HCl and its encapsulated form in Span 60.

Based on the fact that Class III drugs are characterized by low permeability due to their high hydrophilic characters, it was attempted to encapsulate the drug in Span 60 to improve its lipophilicity. The partition coefficient is the parameter that relates the method in which there is a single solute partition between polar (usually water) and nonpolar phases (Leo, 1993). N-octanol is used as nonpolar phase since it is better for simulating the situation in a living tissue. It is similar to the membrane lipid 1-octanol that has a lipophilic long alkyl chain and a polar hydroxyl group (Pagliara et al., 1999). Accordingly, the octanol-water partition coefficient (log P) is the best *in vitro* behavior of a compound toward a membrane. Then, log P can be defined as the logarithm of the ratio of the solute concentration in the octanol phase to the solute concentration in the water phase, at a defined temperature (Rothwell et al., 2005). **Table 4** shows the results of partitioning of pure metformin HCl and Span 60 matrices containing the drug prepared by using different TDC. From the table it can be noticed that Log P value increased markedly by increasing the concentration of Span 60 in the matrix. This result could also be clearly noticed from (**Figure 5**), which emphasizes that this effect is due to molecular dispersion of the metformin HCl in Span 60 as a matrix. Accordingly, it could be reported that the molecular dispersion of the drug in the Span 60 matrix and the marked increasing of its partition coefficient is due to changing the hydrophilic character of the drug molecules to a lipophilic one.

**Table 4:** Log P values of pure drug and that encapsulated in different % of Span 60.

Span 60%	Log P
00%	-0.560
25%	0.042
33%	0.063
50%	0.099
66%	0.109
75%	0.114

**Figure 6:**
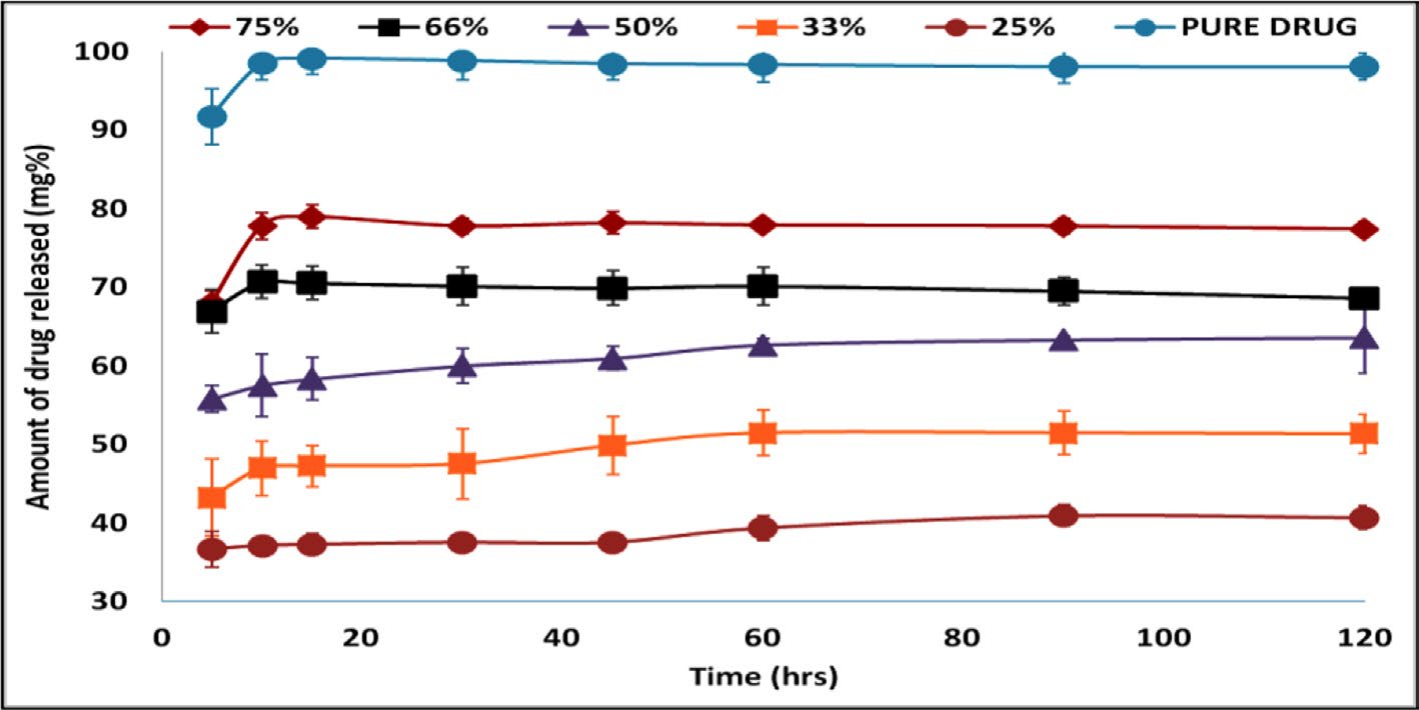
Drug release profile of pure drug and from Span 60 products prepared by using different TDC in phosphate buffer pH 6.8.

**Figure 6** shows the drug release profile from different Span 60 products prepared by using different TDC and dissolution profile of the pure drug. To avoid confusion, it should be noticed that the TDC was used in plotting the drug release profile. The dissolution profile of the pure drug shows rapid and complete dissolution, which is due to high drug solubility in the dissolution medium. Also, from the figure, it is clear that in the dissolution profile of the drug from Span 60 matrices, there is a burst effect and incomplete drug release. These two effects depend on the TDC used. Increasing the TDC used leads to increasing the burst effect and the opposite could be noticed by incomplete drug release. The presence of burst effect is may be due to molecular dispersion of the drug in the matrix which increased by increasing TDC used. Incomplete drug release may be due insolubility of Span 60 in the dissolution medium.

The use of “intestinal sacs” for assaying the permeability is a quick and sensitive technique for determining the overall intestinal integrity or comparative transport of a specific molecule, with the added benefit of intestinal site specificity. The calculation of the apparent permeability (Papp) or permeation coefficient of a molecule through the intestinal barrier could be done. The intestinal sac technique could be used for assaying the absorption of the drug or examining the local epithelial barrier dysfunction in gastrointestinal disease of the animal models (Mateer et al., 2016). Two drawbacks for the using of *in vitro* permeability test were also reported. First, the absence of blood and lymph flow in the isolated intestinal segment, then the *in vivo* permeability, in reality, would be expected to be much higher than the permeability measured *in vitro* (Houston and Wood, 1980; Page and Carlson, 1991). Second, in the *in vitro* model, the roles of the luminal contents and intestinal motility, which may be the major factors in determining the site of intestinal exsorption, are ignored (Page and Carlson, 1991; Rozman et al., l985). The suspension of the prepared segment on the shaft of the USP dissolution apparatus leads to preventing the formation of stagnant diffusion layer around the segment. Therefore, the concentration of the drug in accepted compartment is very low compared to the donor one. This represents the role of blood in the *in vivo* test. In addition, the rotation of the suspended segment creates two forces: centrifugation force for the solution in the donor compartment on the inner wall of the segment and pressing force of the outside medium on the outside wall of the segment. These two forces may represent the intestinal movements. Concerning the lack of the intestinal content which affects the drug permeability, there are no regulations except by fasting for empty intestinal segment or special food consumption, which could also be considered in the experimental design. Accordingly, it can be reported that the idea of the suspension of the intestinal segment may solve the drawbacks of the *in vitro* test.

**Figure 7:**
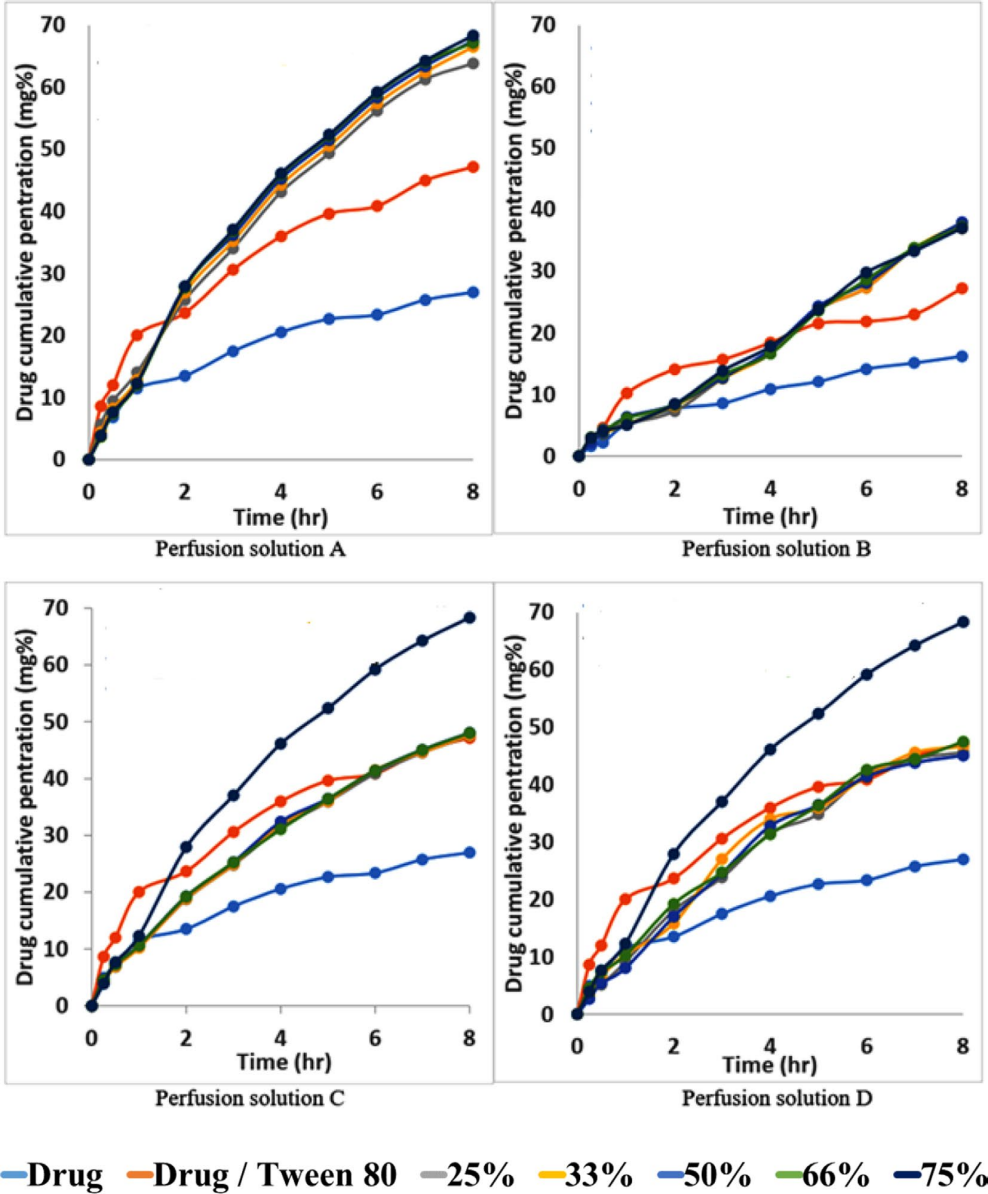
Permeability profile of metformin HCl from intestinal sacs containing different perfusion solution.

**Figures 7A–D** shows the permeability profile of the drug from different permeation solutions. From **Figure 7A**, the markedly increasing effect of the addition of Tween 80 to the drug on the permeation profile of the drug can be noticed. This effect is again greatly increased from Span 60-encapsulated drug particles and emulsified by Tween 80. The pretreatment of the intestinal wall with phosphatidylcholine led to markedly decreasing the drug penetration effect because phosphatidylcholine is a lipophilic barrier for a hydrophilic drug and drugs encapsulated in the dosage form (**Figure 7B**). The physical mixture of the pure Span 60 with drug-encapsulated particles emulsified with Tween 80 (perfusion solution C), or that was melted and then emulsified by Tween 80 (perfusion solution D), showed no difference in the drug permeation profile. These results may indicate that the excess Span 60 as either a pure form or melted with encapsulated drug particles has no effect on the drug permeation process (**Figures 7C, D**).

The permeability parameters of metformin HCl, metformin-Tween 80 and Span 60-encapsulated metformin HCl across the non-everted sac was determined, and the results are summarized in table (Adeyeye and Price, 1994). Normally the *in vitro* permeability parameters are the following: a) lag time, which is the extrapolation of the straight-line part to the x-abscissa in times (Stanley and Stanley, 1972). The lag time could be considered the distribution of the solute in the tissue before the achievement of the steady-state flux of the solute. It was reported that there is a direct relationship between the lipid solubility of the solute and its volume distribution in the tissue. The lag time increases with increasing lipophilicity of the solute and with increasing volume of distribution (Houston and Wood, 1980; Page and Carlson, 1991). Knowledge of both permeability and lag time for permeants provides insight into the potential permeation pathway. Unfortunately, there are not many experimental lag times reported in the literature (Ana et al., 2006). Also, in this manner the extrapolation of the straight-line part of the permeation curve may not be intercepted with the x-abscissa in frequency but with the y-abscissa in concentration. From the mathematic view, the intercept with y-abscissa represents the drug penetration at zero time. In more precise words, it could be regarded as the distribution of the solute in the paracellular pathway tissue before the achievement of the steady-state flux of the solute. There is also b) the total drug penetration percent, which is the cumulative amount of drug transferred. This value could also be used for the comparison of the absorption of the drug from the prepared dosage form to that from the pure drug as a tool to indicate the absorption enhancement effect of the drug carrier (Span 60) and consequently the drug bioavailability. In addition, the percent of drug absorption enhancing (% DAE) could also be calculated from the following equation:

$$\begin{array}{l} \text{\% DAE}\\ \;=\frac{\text{Cummulative amount of drug penetrated from dosage from}}{\text{Cummulative amount of pure drug penetrated}}\times 100 \end{array}$$

From **Table 5**, it can be noticed that the values of **r** **^2^** in each case are high enough to consider good fitting of the data. Also, in each case, there is no lag time, and instead there is a value for the intercept with y-abscissa in concentration (**Table 5A**). The absence of the lag time may be due to high water solubility of the drug which is probably responsible for the intercept values. The intercept value represents, in this case, the rapid saturation of the paracellular pathway tissues of the intestinal wall with the drug before the drug transportation. This result is supported by the fact that about 90% of metformin HCl is absorbed *via* the paracellular pathway (Proctor III, 2010). The addition of Tween 80 to the drug led to a marked increase of all permeation parameters. This is possibly due to the role of Tween 80 on the activity inhibition of protein kinase C (PKC), the decreasing phosphorylation of P-gp, and the modulating P-gp mediated drug efflux should also be considered (Komarov et al., 1996; Yu-li, 2003; Zhang et al., 2003; Al-Mohizea, 2010). The significant saturable components of the paracellular pathway may be also affected by the presence of Tween 80, especially the saturable paracellular pathway mediated by electrostatic interactions between the opposite charges of diffused substances (drug and Tween 80) and the anionic residues of the lateral space and/or tight junctions (Proctor III, 2010).

**Table 5:** Data of metformin HCl Transferred through non-everted intestinal sac (Method one).

Perfusion solution A	r ^2^	Papp	Intercept	Total penetration %	Absorption enhancement %
Metformin	0.971	0.0010	0.0072	26.972 ± 1.21	100.000
Metformin + tween	0.971	0.0018	0.0127	47.159 ± 0.21	174.842
25% Span + tween	0.973	0.0034	0.0103	63.840 ± 0.61	236.687
33% Span + tween	0.984	0.0035	0.0105	66.520 ± 0.13	246.625
50% Span + tween	0.984	0.0035	0.0110	67.495 ± 0.22	250.238
66% Span + tween	0.977	0.0034	0.0117	67.143 ± 0.28	248.935
75% Span + tween	0.984	0.0035	0.0115	68.349 ± 0.35	253.404
**Perfusion solution B**					
Metformin	0.988	0.0008	0.0026	16.212 ± 0.80	100.000
Metformin + tween	0.963	0.0012	0.0057	27.233 ± 0.36	167.978
25% Span + tween	0.995	0.0029	-0.0015	36.933 ± 1.36	227.808
33% Span + tween	0.996	0.0029	-0.0014	37.838 ± 1.10	233.387
50% Span + tween	0.997	0.0029	-0.0011	37.959 ± 1.35	234.136
66% Span + tween	0.994	0.0028	-0.0011	37.224 ± 0.56	229.599
75% Span + tween	0.994	0.0028	-0.0005	37.069 ± 0.41	228.643
**Perfusion solution C**					
Metformin	0.971	0.0010	0.0072	26.972 ± 1.21	100.000
Metformin + tween	0.971	0.0018	0.0127	47.159 ± 0.21	174.842
25% Span + tween	0.984	0.0027	0.006	47.523 ± 0.27	176.193
33% Span + tween	0.980	0.0027	0.0061	47.637 ± 0.29	176.616
50% Span + tween	0.982	0.0027	0.0064	48.058 ± 0.58	178.175
66% Span + tween	0.987	0.0028	0.0061	48.009 ± 0.69	177.993
**Perfusion solution D**					
Metformin	0.971	0.0010	0.0072	26.972 ± 1.21	100.000
Metformin + tween	0.971	0.0018	0.0127	47.159 ± 0.21	174.842
25% Span + tween	0.970	0.0033	0.0026	45.623 ± 0.69	169.149
33% Span + tween	0.961	0.0033	0.0029	46.673 ± 1.89	173.040
50% Span + tween	0.958	0.0033	0.0024	45.076 ± 0.53	167.119
66% Span + tween	0.977	0.0032	0.0033	47.496 ± 1.77	176.092

The permeation parameters (permeation coefficient, total penetration% and DAE%) are again markedly increased from the Span 60-encapsulated drug (**Table 5**), with slightly decreased intercept values compared to those from tween alone. In this study it was found that the partition coefficient of metformin HCL is largely increased *via* its encapsulation in Span 60, indicating the drug entrapped in the polar part of the surfactant. This suggested entrapment process led to decreasing the drug release process in phosphate buffer due to insolubility of Span 60 in the dissolution media. Emulsification of Span 60-encapsulated drug in perfusion solution (phosphate buffer pH 6.8) by using Tween 80 indicated the change of the surface again to hydrophilic. This image may be responsible for the above permeation parameter results for two reasons: first, the addition of Tween 80 to emulsify the Span 60-encapsulated drug may correct the HLP value (hydrophilic-lipophilic balances) of the image to be easily diffused. Second, the addition of Tween 80 may lead to increasing the size of the image, which leads to decreasing the value of intercept (rapid saturation of the tissue). The suggested hypothesis may be supported by the novel “sponge” hypothesis, which was formulated to explain how metformin could be highly absorbed across human intestines through predominantly a paracellular mechanism (Proctor III, 2010).

As evidence for the above explanation, the intestinal segments were pretreated with phosphatidylcholine. Pretreatment of the segments with phosphatidylcholine (perfusion solution B) led to a marked decrease in the intercept values of metformin and very small lag time from Span 60-encapsulated drugs. This is due to increasing the intestinal surface hydrophobicity by phosphatidylcholine (Dial et al., 2008). At the same time, the presence of tween with drug alone led to increasing the intercept value compared to the others due to the emulsifying effect of Tween 80 on the intestinal-protected layer by phosphatidylcholine. The decrease in the total permeation coefficient values are not parallel to the intercept values, indicating that once the image diffuses the protected layer, they diffuse again with the previously suggested pathway. The similarity in the DAE% to that without a protective phosphatidylcholine layer was because the cumulative amount of drug penetrated with protected phosphatidylcholine was used in the calculation. In addition, these results supported what was stated before about the decreasing effect, which was due to the presence of protective layer.

The results of the addition of pure Span 60-encapsulated drug as a physical mixture (perfusion solution C) showed a decrease in the values of intercept from both the drug itself and the drug with tween, with a slight decrease in the permeation coefficient. This is due to the expected diffusion compatibility between the emulsified Span 60-encapsulated drug and the emulsified Span 60, which was added as pure form. This diffusion competition led also to decreasing the total penetration and DAE percent. To prove the role of addition of pure span as a physical mixture on the drug transfer process, other physical mixtures were prepared, melted to produce clear solution, cooled, and the resulted masses were used for perfusion solution D preparation. From **Table 5D**, it can be noticed that melting the physical mixture before emulsification in the perfusion solution D led to increasing the permeation coefficient to that from Span 60 particles but decreasing the intercept values. Decreasing the intercept values may be due to increasing the size of the image suggested and in agreement with the novel sponge hypothesis, which was formulated to explain how metformin could be highly absorbed across the human intestines through predominantly a paracellular mechanism (Proctor III, 2010). Bringing the permeation coefficient to that from Span 60-encapsulated particles may indicate the role of molecular dispersion of the drug in Span 60 for diffusion and that it is a saturable one.

Applying the second reported calculation method from **Table 6**, noticed some changes in the calculation results could be noticed. Studying the results led to the same conclusion reported above.

**Table 6:** Data of metformin HCl Transferred through non-everted intestinal sac (Method two).

Perfusion solution A	r ^2^	Papp	Intercept	Total penetration %	Absorption enhancement %
Metformin	0.971	01.7 × 10^-5^	12.787	26.972 ± 1.21	100.000
Metformin + tween	0.971	2.99 × 10^-5^	22.386	47.159 ± 0.21	174.842
25% Span + tween	0.973	5.66 × 10^-5^	18.250	63.840 ± 0.61	236.687
33% Span + tween	0.984	5.87 × 10^-5^	18.502	66.520 ± 0.13	246.625
50% Span + tween	0.984	5.87 × 10^-5^	19.477	67.495 ± 0.22	250.238
66% Span + tween	0.977	5.73 × 10^-5^	20.744	67.143 ± 0.28	248.935
75% Span + tween	0.984	5.87 × 10^-5^	20.331	68.349 ± 0.35	253.404
**Perfusion solution B**					
Metformin	0.988	1.41 × 10^-5^	4.6003	16.212 ± 0.80	100.000
Metformin + tween	0.963	1.93 × 10^-5^	10.004	27.233 ± 0.36	167.978
25% Span + tween	0.995	4.77 × 10^-5^	–2.7002	36.933 ± 1.36	227.808
33% Span + tween	0.996	4.78 × 10^-5^	–2.4368	37.838 ± 1.10	233.387
50% Span + tween	0.997	4.76 × 10^-5^	–2.0312	37.959 ± 1.35	234.136
66% Span + tween	0.994	4.71 × 10^-5^	–1.8962	37.224 ± 0.56	229.599
75% Span + tween	0.994	04.6 × 10^-5^	–0.9662	37.069 ± 0.41	228.643
**Perfusion solution C**					
Metformin	0.971	01.7 × 10^-5^	12.787	26.972 ± 1.21	100.000
Metformin + tween	0.971	2.99 × 10^-5^	22.386	47.159 ± 0.21	174.842
25% Span + tween	0.984	4.56 × 10^-5^	12.615	47.523 ± 0.27	176.193
33% Span + tween	0.980	4.56 × 10^-5^	10.861	47.637 ± 0.29	176.616
50% Span + tween	0.982	4.55 × 10^-5^	11.299	48.058 ± 0.58	178.175
66% Span + tween	0.987	4.59 × 10^-5^	10.832	48.009 ± 0.69	177.993
**Perfusion solution D**					
Metformin	0.971	01.7 × 10^-5^	12.787	26.972 ± 1.21	100.000
Metformin + tween	0.971	2.99 × 10^-5^	22.386	47.159 ± 0.21	174.842
25% Span + tween	0.970	5.45 × 10^-5^	4.552	45.623 ± 0.69	169.149
33% Span + tween	0.961	5.50 × 10^-5^	5.203	46.673 ± 1.89	173.040
50% Span + tween	0.958	5.4 × 10^-5^	4.329	45.076 ± 0.53	167.119
66% Span + tween	0.977	5.38 × 10^-5^	5.876	47.496 ± 1.77	176.092

## Conclusion

This study suggested a new promising solution for the low permeability of class III drugs. This solution is based on molecular dispersion of the drug in Span 60 as a drug encapsulation matrix. The microspheres or microparticles of Span 60 as a matrix could prepared by either a solvent evaporation technique or a melting congealing technique. The technique selected will be based on the solubility of the drug in the organic phase. The rapid congealing of the melted drug-Span 60 phase represents a good solution to increase the drug entrapment in the Span 60 microsphere matrix. Considering the emulsification system of the gastrointestinal tract, which will emulsify the Span 60-encapsulated drug matrix instead of the Tween 80 used in the study, a huge improvement of class III permeability and consequently bioavailability could be expected. In addition, this study may also open the door to using the same technique to enhance the drug absorption mechanisms by the paracellular pathway for rapid and complete pharmacological effect.

## Data Availability

The raw data supporting the conclusions of this manuscript will be made available by the authors, without undue reservation, to any qualified researcher.

## Ethics Statement

The experimental protocol has been approved by the College of Pharmacy Ethical Committee.

## Author Contributions

All the authors have contributed equally to the manuscript, and have reviewed and approved the submission.

## Conflict of Interest Statement

The authors declare that the research was conducted in the absence of any commercial or financial relationships that could be construed as a potential conflict of interest.
